# Characterization of the Antinociceptive Activity from *Stevia serrata* Cav

**DOI:** 10.3390/biomedicines8040079

**Published:** 2020-04-07

**Authors:** Millena S. Cordeiro, Daniel L. R. Simas, Juan F. Pérez-Sabino, Max S. Mérida-Reyes, Manuel A. Muñoz-Wug, Bessie E. Oliva-Hernández, Antônio J. R. da Silva, Patricia D. Fernandes, Thais B. S. Giorno

**Affiliations:** 1 Institute of Biomedical Sciences, Federal University of Rio de Janeiro, Rio de Janeiro 21941-902, Brazil; 2 Institute of Natural Products Research, Federal University of Rio de Janeiro, Rio de Janeiro 21941-902, Brazil; 3 School of Chemistry, Faculty of Chemical Sciences and Pharmacy, University of San Carlos of Guatemala, Guatemala 01012, Guatemala

**Keywords:** *Stevia serrata*, essential oil, inflammation, antinociception, pain

## Abstract

Background: *Stevia serrata* Cav. (Asteraceae), widely found in Guatemala, is used to treat gastrointestinal problems. The aim of this study was to demonstrate the antinociceptive and anti-inflammatory effects of the essential oil (EO) and the mechanism of action. Methods: EO was tested in chemical (capsaicin- and glutamate-induced licking response) or thermal (hot plate) models of nociception at 10, 30 or 100 mg/kg doses. The mechanism of action was evaluated using two receptor antagonists (naloxone, atropine) and an enzyme inhibitor (L-NAME). The anti-hyperalgesic effect was evaluated using carrageenan-induced nociception and evaluated in the hot plate. Results: All three doses of EO reduced licking response induced by glutamate, and higher doses reduced capsaicin-induced licking. EO also increased area under the curve, similar to the morphine-treated group. The antinociceptive effect induced by EO was reversed by pretreatment of mice with naloxone (1 mg/kg, ip), atropine (1 mg/kg, ip) or L-NAME (3 mg/kg, ip). EO also demonstrated an anti-hyperalgesic effect. The 100 mg/kg dose increased the latency time, even at 1 h after oral administration and this effect has been maintained until the 96th hour, post-administration. Conclusions: Our data suggest that essential oil of *S. serrata* presents an antinociceptive effect mediated, at least in part, through activation of opioid, cholinergic and nitrergic pathways.

## 1. Introduction

*Stevia serrata* Cav. is a plant of the Asteraceae family (Asteroideae) that grows in Central America and Mexico, usually over 1500 m, and in northern South America at higher altitudes. In Guatemala it is found in the regions of Chimaltenango, Huehuetenango, El Quiche, Sacatepéquez and Sololá, near pine and oak forests in sunny sites [1]. This plant grows as a perennial herb, from 0.6–1 m tall, with stems puberulent to densely pilose, linear-spatulate to oblanceolate leaves, an apex rounded to acute, 2–6 cm long and 0.2–1.5 cm wide blades [2]. This plant has the following synonymia: *Ageratum punctatum* Ortega, *Stevia ivifolia* Willd., *Stevia pubescens* Kunth, *Stevia punctata* (Ortega) Pers., *Stevia serrata* var. *ivifolia* (Willd.) B.L. Rob., *Stevia virgata* Kunth [2].

Recently, it has been demonstrated that chamazulene, a sesquiterpene, is the major component of the essential oil (60.1%) and suggested that essencial oil (EO) reduced the time that mice spent licking the formalin-injected paw [3]. However, in the paper, neither the possible effects of the EO in other models of nociception, nor the mechanism of action was studied. In this regard, the aim of the present paper was to evaluate the antinociceptive effect of EO in other models of nociception, i.e., capsaicin- and glutamate-induced licking, hot plate and carrageenan-induced hyperalgesia, and to identify the mechanism by which *S. serrata* exerts its effect.

## 2. Materials and Methods

### 2.1. Plant Material and Extraction

Aerial parts of *S. serrata* were collected in September 2014, from a population found in San José Chacayá, province of Sololá, west from Guatemala City. A voucher specimen was kept at the Herbarium of the Faculty of Chemistry and Pharmacy of the University of San Carlos, Guatemala (BIGU 72832). The oil from 40 g of aerial parts of *S. serrata* was extracted by hydro distillation using a clevenger-type apparatus for 2 h. A yield of 0.2% (*w*/*w*) was obtained. Essential oil (EO) was maintained at −20 °C until use.

### 2.2. Essential Oil Chemical Composition

The essential oil was analyzed by gas chromatography/-mass spectrometry (GC-MS) according to Simas et al. (2017). The identification of the EO components was made by comparison of their mass spectra and retention indexes with data from the literature [4]. The compounds found in higher concentrations were the sesquiterpenes chamazulene (60.1%), (*E*)-nerolidol (7.3%), caryophyllene oxide (6.3%) and germacrene D (5.4%).

### 2.3. Animals

Swiss *Webster* mice (20–25 g, 8–10 weeks, 200 animals) of both sexes were donated by the Institute Vital Brazil (Niteroi, RJ, Brazil). Animals have been housed in a temperature-controlled room at 22 ± 2 °C with a 12 h light/dark cycle and free access to pelleted food (Nutrilab, Belo Horizonte, MG, Brazil) and water. Twelve hours before each experiment, the animals received only water in order to avoid food interference with substance absorption. The experimental protocols used in this work followed the rules advocated by Law 11,794, from October 8th 2008 by the National Council of Animal Experimentation Control (CONCEA) and were approved by the Ethics Committee of Animal Use (CEUA), Science Centre Health/UFRJ (DFBCICB015-04/16).

### 2.4. Drugs, Reagents and Treatments

All solvents were chromatographic grade (Tedia, Rio de Janeiro, RJ, Brazil). Carrageenan, glutamic acid, atropine, Nω-nitro-L-arginine methyl ester (L-NAME) were purchased from Sigma-Aldrich (St. Louis, MO, USA). Formalin was purchased from Merck (Darmstadt, Germany). Cristália (São Paulo, Brazil) kindly provided morphine sulphate and naloxone hydrochloride. Capsaicin was purchased from Galena (Campinas, SP, Brazil). A stock solution at 100 mg/mL in extrapure oil was prepared with the essential oil (EO). This EO was administered to mice by oral gavage, at doses of 10 to 100 mg/kg, in a final volume of 0.1 mL, 60 min prior to experiments. Morphine (5 mg/kg, p.o.) was diluted in extrapure oil just before use and was used as a reference drug. The control group received vehicle (extrapure oil) by oral gavage.

### 2.5. Capsaicin- and Glutamate-Induced Nociception

Animals received oral administration of EO (10, 30 or 100 mg/kg) one hour before intraplantar injection of capsaicin (20 μL, 1.6 μg/paw). Mice were individually placed in a transparent glass observation chamber. Based on Giorno et al. [5], nociception was assessed immediately after injection and quantified by paw licking time during a period of 5 min.

In the glutamate-induced licking test, the mice were orally treated with the EO (10, 30 and 100 mg/kg), 60 min before intraplantar injection of glutamate (20 μL, 3.7 ng/paw). Immediately after the injection, the animals were individually placed in a transparent glass observation chamber. The nociception was considered as the total time (recorded with a chronometer) the animals remained licking the injected paw [5].

### 2.6. Formalin-Induced Nociception

This assay was performed as described by Sakurada et al. [6] and adapted by Giorno et al. [5]. After an intraplantar injection of formalin (20 μL, 2.5% *v*/*v*), the period during which mice remained licking the injected paw was immediately recorded. This response has been divided in two phases: The first one, between the injection and 5 min (neurogenic phase) and the second one, between 15–30 min post-formalin injection (inflammatory phase). EO or vehicle was administered 60 min before the injection of formalin.

### 2.7. Hot Plate Test

According to the method described previously [7] and adapted by Matheus et al. [8], the animals were placed in a glass cylinder on a heated metal plate maintained at 55 ± 1 °C every 30 min after administration of EO (10, 30 and 100 mg/kg) until 180 min. The latency of nociceptive responses, such as jumping or licking of the hind paws, was recorded with a stopwatch. Two measurements were taken 30 and 60 min before the treatment of animals and the average of these measurements was referred to as “baseline”.

### 2.8. Thermal Hyperalgesia

The methodology described by Sammons et al. [9], with some modifications, was used. Briefly, the hyperalgesia was induced by carrageenan (2%, 25 µL) injection in the right hind paw, 30 min after oral treatment with EO (10, 30 and 100 mg/kg) or vehicle. The animals were individually placed in a hot plate apparatus (55 ± 1 °C). At intervals of 1, 2, 4, 6, 24, 48, 72 and 96 h after the treatment, the time period (in seconds) necessary for animals to jump or lick the carrageenan-injected paw was recorded.

### 2.9. Mechanism of Action

For the study of the possible mechanism of action of *S. serrata*, mice received intraperitoneal injection of naloxone (a non-selective opioid receptor antagonist, 1 mg/kg), atropine (non-selective muscarinic receptor antagonist, 1 mg/kg) or L-NAME (inhibitor of nitric oxide synthase enzyme, 3 mg/kg) 15 min prior to oral administration of *S. serrata* EO (100 mg/kg). Antinociception was evaluated in the hot plate test, as previously described (Section 2.7.). The doses of antagonists and inhibitor were chosen based on previous data described in the literature [10,11]. The experiments conducted in our laboratory and dose response curves for each antagonist were previously constructed, and the dose that reduced 50% of the responses of the agonist was chosen for these assays [12,13].

### 2.10. Locomotor Performance and Spontaneous Activity Evaluation

To exclude a possible central effect, both the spontaneous activity and the locomotor performance have been evaluated as adapted by Barros et al. [14]. Each animal received oral administration of *S. serrata* EO (100 mg/kg). They have been immediately placed in a chamber with the floor divided into 50 squares (5 cm × 5 cm). The total number of squares in which mice walked has been counted. For locomotor evaluation, mice were trained in apparatus (rotarod; 3.7 cm in diameter, 8 r.p.m) until they remained in for 60 s without falling. On the day of the experiment, mice were treated with EO (100 mg/kg) and the total number of falls was recorded. In both protocols, mice were evaluated at 30, 60, 150 and 240 min after administration.

### 2.11. Statistical Analysis

Each group was composed by 6 animals, randomly divided. The results are presented as the average ± standard deviation (S.D.). Statistical analyses were performed using analysis of variance (ANOVA) followed by Bonferroni test using Prism Software 5.0 (Graph-Pad Software, La Jolla, CA, USA). The p values of 0.05 have been considered as indicative of significance.

## 3. Results

### 3.1. Effect of Essential Oil of Stevia Serrata on Capsaisin and Glutamate Induced-Licking

Previous results from our group indicated that EO from *S. serrata* reduced formalin-induced licking response in a dose response manner, to doses of 10, 30 and 100 mg/kg [3]. In view of these previous results, we decided to further investigate whether EO from *S. serrata* could present a central antinociceptive effect, and the possible mechanism of action.

**Figure 1:**
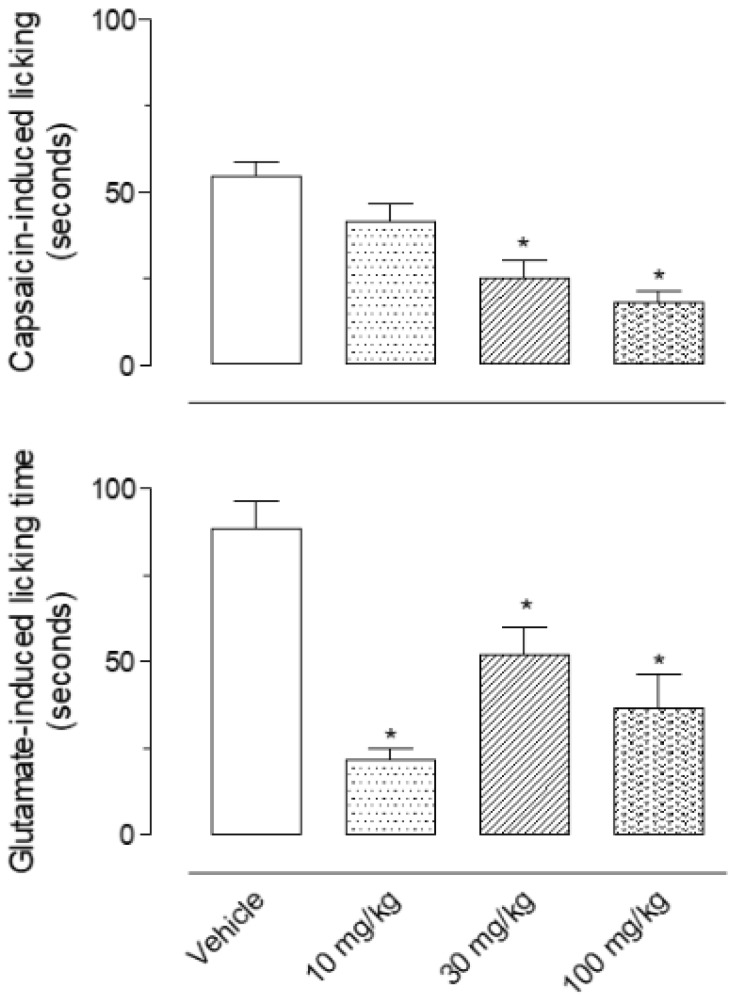
Effect of essential oil of *Stevia serrata* in the capsaicin- and glutamate-induced licking response. The animals have been orally pretreated with the vehicle or essential oil (10, 30, 100 mg/kg) 1 h before the injection of capsaicin (1.6 μg/paw) or glutamate (3.7 ng/paw). Results are expressed as mean ± S.D. (*n* = 6). Data have been analyzed by ANOVA, followed by Bonferroni post-test, * *p* < 0.05 has been considered as significant when compared to the vehicle-treated groups.

Figure 1 shows the nociception after capsaicin or glutamate intraplantar injection and the effects observed after pretreatment of mice with increasing doses of EO. It could be noted that 30 and 100 mg/kg doses of EO significantly reduced licking induced by capsaicin (56.8% and 68.7% of inhibition, respectively) while all three doses (10, 30 and 100 mg/kg) reduced the response induced by glutamate (75.4%, 41.3% and 58.7% inhibition, to 10, 30 and 100 mg/kg, respectively).

### 3.2. Effect of Essential Oil of Stevia Serrata on the Hot Plate Test

**Figure 2:**
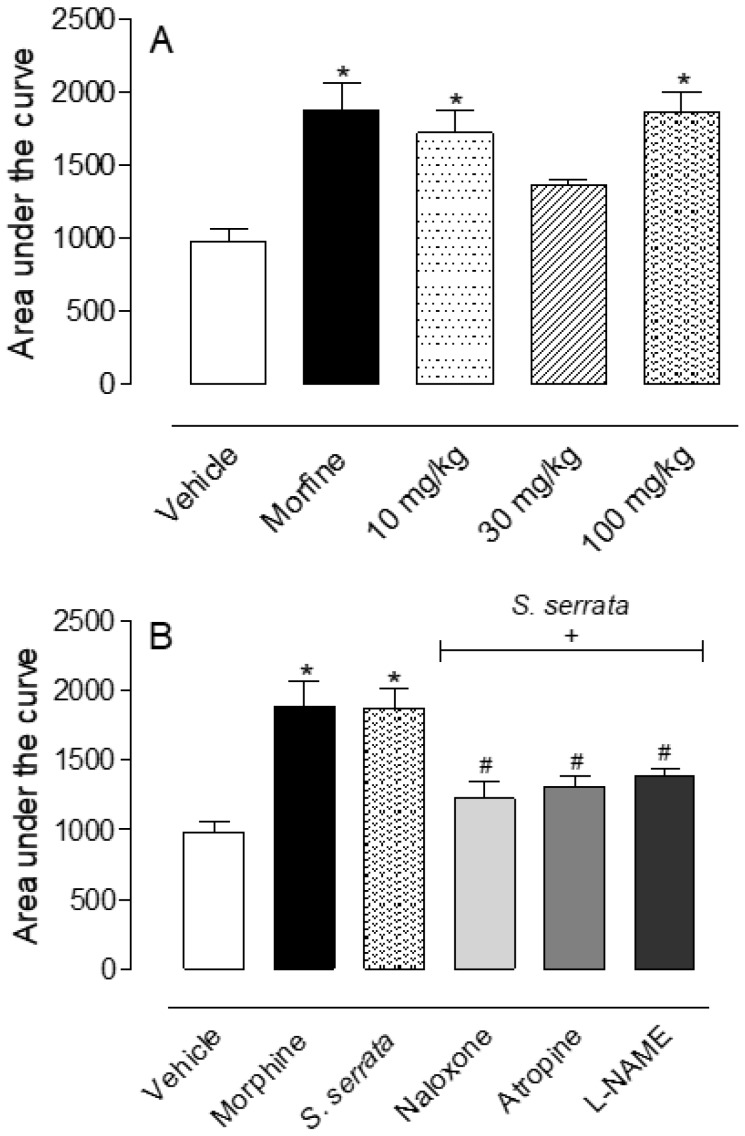
Effects of essential oil of *Stevia serrata* and different antagonists in the thermal nociception model (hot plate). The mice were pretreated orally with the vehicle, essential oil (10, 30, 100 mg/kg) or morphine (2.5 mg/kg) and nociceptive effect was evaluated in the hot plate model (**A**). The animals have been pretreated with naloxone (1 mg/kg, i.p.), atropine (1 mg/kg, i.p.) or L-NAME (3 mg/kg, i.p.) 15 min before oral administration of EO (100 mg/kg) or vehicle (**B**). Results are expressed as mean ± S.D. (*n* = 6) of area under the curve calculated by GraphPad Prism Software 5.0. Data have been analyzed by ANOVA, followed by Bonferroni post-test. * *p* < 0.05 has been considered as significant when compared to the vehicle-treated group and ^#^ *p* < 0.05 when comparing with *S. serrata*-treated group.

We also evaluated if EO could present central antinociceptive activity using the hot plate test. Increasing doses of orally administered EO presented antinociceptive activity, similar to data obtained after pretreatment of mice with morphine (an opioid agonist), the positive control drug. Values of area under the curve obtained with all doses of EO varied between 1500 and 2000 arbitrary units, and after morphine, pretreatment values were almost 2000 (Figure 2A). To investigate the possible mechanism of antinociception induced by *S. serrata* essential oil, mice have been pretreated with naloxone (an opioid receptor antagonist, 1 mg/kg, i.p.), atropine (a cholinergic receptor antagonist, 1 mg/kg, i.p) or L-NAME (inhibitor of nitric oxide synthase enzyme, 3 mg/kg, i.p.) 15 min before oral administration of EO (100 mg/kg). Data in Figure 2B shows that both the antagonists, naloxone and atropine, as well the enzyme inhibitor partially reversed the effect caused by EO and reduced its antinociceptive activity in almost 50% of cases.

### 3.3. Effect of Essential Oil of Stevia Serrata on Formalin Induced-Licking

**Figure 3:**
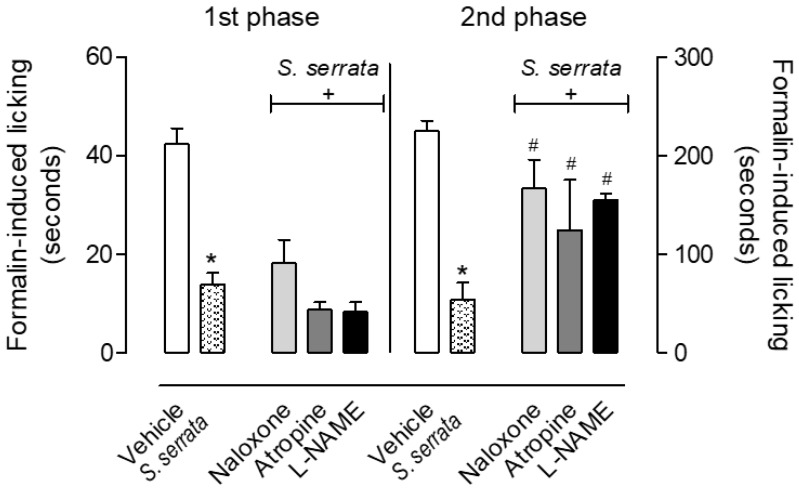
Effects of different antagonists on the antinociceptive activity of the essential oil of *Stevia serrata* in the formalin-induced licking response. Mice received intraperitoneal injection of naloxone (1 mg/kg), atropine (1 mg/kg) or L-NAME (3 mg/kg) 15 min prior to oral administration with the vehicle or essential oil (100 mg/kg). After 60 min, mice received an intraplantar injection of formalin (20 µL, 2.5%). Results are expressed as mean ± S.D. (*n* = 6). Data have been analyzed by ANOVA, followed by Bonferroni post-test. * *p* < 0.05 has been considered as significant when compared to the vehicle-treated groups and ^#^ *p* < 0.05 when comparing with *S. serrata*-treated group.

Sequentially, whether the same antagonists would also have activity in the formalin-induced licking response was also evaluated. As can be observed in Figure 3, none of the antagonists and enzyme inhibitors demonstrated an effect in the first phase of the licking response. However, all three drugs almost completely reversed the antinociceptive effect of EO in the second phase of the model.

### 3.4. Effect of Essential Oil of Stevia Serrata in the Thermal Hyperalgesia Model

**Figure 4:**
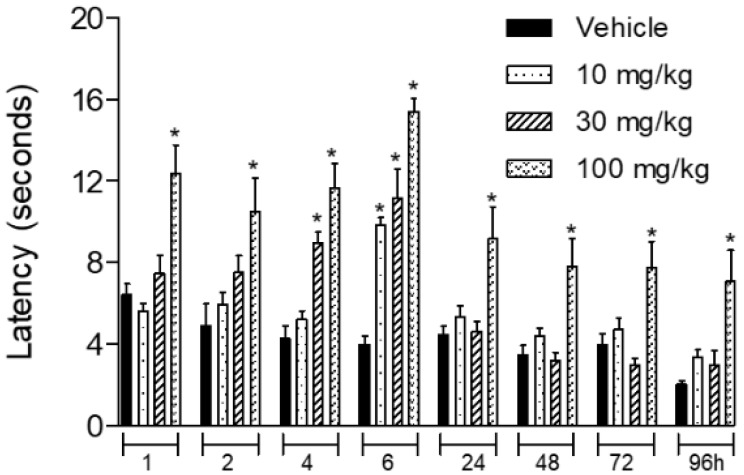
Effect of essential oil of *Stevia serrata* in the hyperalgesic effect induced by carrageenan. The animals have been pretreated orally with the vehicle or essential oil (10, 30, 100 mg/kg) 1 h before intraplantar injection of carrageenan (1%/paw). Hyperalgesia has been evaluated in the hot plate model. Results are expressed as mean ± S.D. (*n* = 6). Data have been analyzed by ANOVA, followed by Bonferroni post-test, * *p* < 0.05 has been considered as significant when compared to the vehicle-treated groups.

As the essential oil of *Stevia serrata* presented a significant antinociceptive effect in the inflammatory (formalin-induced licking) and thermal models (hot plate) of nociception, we further decided to analyze if it could present activity in a model of hyperalgesia. In this regard, carrageenan was injected in the paws of mice previously treated with increasing doses (10, 30 or 100 mg/kg) of the essential oil. As the time passes after intraplantar injection of carrageenan, a reduction of latency time could be observed. Even at 96 h post-carrageenan injection, a reduction in latency time could be observed. At the 4th hour after oral treatment, higher doses (30 and 100 mg/kg) of EO significantly increased the latency time. And at the 6th hour, all three doses presented capacity in increasing the period necessary for animals to respond to the hyperalgesic stimulus. It is important to report that the dose of 100 mg/kg presented a significant anti-hyperalgesic effect during the entire assay. Increased latency time was observed from 1 to 96 h post-oral administration of EO (at 100 mg/kg) (Figure 4).

## 4. Discussion

In the present work, it has been demonstrated that the essential oil obtained from aerial parts of *Stevia serrata* presents significant antinociceptive activity in thermal (hot plate) and capsaicin and glutamate-induced licking. It has also been demonstrated that these effects are partially mediated through opioid, muscarinic and nitrergic pathways.

The effect of *S. serrata* against glutamate and capsaicin-induced algesia is of great interest because both agonists play an important participation in central and peripheral nociceptive processes [15,16,17,18]. Glutamate is the main mediator of excitatory synaptic transmission in the central nervous system and activates several intracellular events, such as alteration in intracellular calcium levels, activation of cellular mediators and opening of ion channels [18,19]. It also induces the release of excitatory amino acids, PGE2, NO and kinins [6,20] and promotes the activation of sensitive fibers that induce the release of several substances in the dorsal horn, which can also activate the TRPV1 receptor in the spinal cord [19,21]. Capsaicin is an agonist of vanilloid receptor type-1 (TRPV1) receptors and can activate nociceptive fibers [22]. The activation of TRPV1 receptors is also mediated by the release of neurotransmitters (i.e., glutamate and substance P), an effect that can participate in nociceptive processing [23,24]. *S. serrata* EO significantly reduced the licking time induced by glutamate and capsaicin. Results with EO against capsaicin- and glutamate-induced nociception corroborate each other. These findings suggest that, at least part of the antinociceptive effect of EO is mediated by the glutamatergic pathway. We can also infer that TRPV1 receptors could be involved, thus contributing to the modulation of the antinociceptive effect of EO.

Our data of capsaicin and glutamate-induced licking can complete previous results from our group in formalin-induced-licking. This model is a biphasic model with involvement of a neurogenic pain (first phase) and inflammatory pain (second phase) [25]. EO from *S. serrata* reduced both phases of this model suggesting the involvement of inflammatory mediators as well as algesic pathways. Therefore, reduction previously observed in formalin-induced licking could be due, at least in part, to a blockage in TRPV1 and/or glutamate receptors.

It has also been demonstrated that naloxone partially reverted the antinociceptive effect of *S. serrata*. Naloxone is an antagonist of opioid receptors, widely distributed in the body. Activation of these receptors by its agonist, morphine, induces several effects, analgesia being one of the most prominent [26,27]. It is possible that different substances present in the EO can act in different pathways acting together amplifying the antinociceptive response.

EO also increased the time period of response in the carrageenan-induced hyperalgesia. It is well known that carrageenan is a phlogistic agent that induces mouse paw inflammation with a biphasic profile. Its response includes a first peak at the 4th hour and a second one at 72 h post-injection. Phase one and phase two were mediated by migration of neutrophils and lymphocytes, respectively, and with liberation of several mediators [28]. It can explain the fact that all three doses of EO increased the time period of response at the 6th hour after treatment. During this period there is an increase in inflammatory mediators (i.e., histamine, prostaglandins) induced by carrageenan in mouse paws. The diversity of substances that can be found in the essential oil may be acting by inhibiting different mediators that are liberated in the paw. The sum of the effects produces an increase in the antinociceptive response.

In this study the oral administration of EO did not affect motor performance evaluated by either forced locomotion in the rotarod or spontaneous locomotion in the open-field test. Thus, the possibility that the antinociceptive effect of the compounds tested is due to any degree of motor impairment or sedation is very low.

It has been previously reported that the compounds found in higher concentration of EO were the sesquiterpenes chamazulene (60.1%), (*E*)-nerolidol (7.3%), caryophyllene oxide (6.3%) and germacrene D (5.4%) [3]. The concentration of chamazulene found in this EO was almost 10 times higher than in oil of chamomile flowers [29,30]. As observed in chamomile, chamazulene is formed during the steps of the essential oil production, being an artifact. The precursor of chamazulene in chamomile is matricin, a sesquiterpene, which suffers a fast degradation to chamazulene via the intermediate chamazulene carboxylic acid [31]. Calderon et al. [32] reported the formation of chamazulene in the course of the column chromatographic separation of the pro-chamazulene components from *Stevia serrata* Cav. of silica gel column. Safayhi et al. [33] studied the effect of chamazulene on the leukotriene production in neutrophilic granulocytes and demonstrated that chamazulene inhibited the formation of leukotriene B4 in intact cells and in the supernatant fraction in a concentration-dependent manner. The second most abundant component in the OE is nerolidol that exhibits antinociceptive and anti-inflammatory activity, involving the GABAergic system and proinflammatory cytokines [34]. On the other hand, it is well known that caryophyllene oxide presents anti-inflammatory and antinociceptive effects [35,36,37]. Thus, the anti-inflammatory effect observed in the present work can be explained, at least in part, by the presence of cariophyllene oxide, chamazulene and nerolidol. It is well known that this sesquiterpene presents anti-inflammatory and antinociceptive effects [35,36,37,38], thus suggesting the effect of the EO tested.

## 5. Conclusions

To the best of our knowledge, this paper is the first to suggest the possible mechanism of action of the essential oil of *Stevia serrata* Cav. and demonstrate its antinociceptive activity.
